# Memristor-Based Spiking Neuromorphic Systems Toward Brain-Inspired Perception and Computing

**DOI:** 10.3390/nano15141130

**Published:** 2025-07-21

**Authors:** Xiangjing Wang, Yixin Zhu, Zili Zhou, Xin Chen, Xiaojun Jia

**Affiliations:** 1 School of Physics and Electronic Engineering, Shanxi Key Laboratory of Wireless Communication and Detection, Shanxi University, Taiyuan 030006, China; zhouzili@sxu.edu.cn (Z.Z.); chenxin5@sxu.edu.cn (X.C.); 2 Yongjiang Laboratory, Ningbo 315201, China; yixin-zhu@ylab.ac.cn

**Keywords:** threshold-switching memristors, spiking neuron circuits, neuromorphic perception systems, brain-inspired computing

## Abstract

Threshold-switching memristors (TSMs) are emerging as key enablers for hardware spiking neural networks, offering intrinsic spiking dynamics, sub-pJ energy consumption, and nanoscale footprints ideal for brain-inspired computing at the edge. This review provides a comprehensive examination of how TSMs emulate diverse spiking behaviors—including oscillatory, leaky integrate-and-fire (LIF), Hodgkin–Huxley (H-H), and stochastic dynamics—and how these features enable compact, energy-efficient neuromorphic systems. We analyze the physical switching mechanisms of redox and Mott-type TSMs, discuss their voltage-dependent dynamics, and assess their suitability for spike generation. We review memristor-based neuron circuits regarding architectures, materials, and key performance metrics. At the system level, we summarize bio-inspired neuromorphic platforms integrating TSM neurons with visual, tactile, thermal, and olfactory sensors, achieving real-time edge computation with high accuracy and low power. Finally, we critically examine key challenges—such as stochastic switching origins, device variability, and endurance limits—and propose future directions toward reconfigurable, robust, and scalable memristive neuromorphic architectures.

## 1. Introduction

Edge-AI workloads, such as 4 K/60 fps video (≈12 Gb/s) or sub-10 ms-latency drone navigation [1,2,3,4], expose fundamental von Neumann limits: memory-compute separation, data-locality loss, and steep energy scaling. Repeated crossings of the “memory wall” raise both latency and power to unsustainable levels [5,6,7,8,9]. With transistor scaling slowing, alternative paradigms are required. The human brain—operating complex cognition below 20 W—achieves low-energy throughput via massive parallelism, event-driven spikes, and in situ memory–compute fusion [10,11,12,13]. Neuromorphic engineering carries these biological insights to electronics. Yet the latest mixed-signal CMOS platforms—Intel Loihi 2, IBM TrueNorth—still dissipate 10–100 pJ spike^−1^ and require millimeter-scale silicon per 10^3^ neurons, three orders of magnitude less dense than cortex [14,15,16]. Threshold-switching memristors (TSMs) overcome this gap by offering intrinsic sub-pJ spiking, <30 ns latency, and nanoscale footprints compatible with ≥10^10^ neurons cm^−2^ [17,18,19,20,21].

Their two-terminal structure further facilitates seamless integration into neuromorphic systems. Volatile TSMs underpin both (i) the device-level emulation of diverse Neuronal firing behaviors—regular, bursting, adaptive, and stochastic—and (ii) system-level sensory front-ends that transduce the light, pressure, or gas concentration directly into spike trains [22,23,24]. Particularly in SNN architectures, memristors can physically emulate key neuronal behaviors such as signal integration, threshold-triggered firing, and reset dynamics [25,26,27]. Their utility extends across both single-neuron behavior modeling and system-level sensory computing. Memristor-based neuron circuits can replicate diverse temporal firing behaviors observed in biological neurons—including regular spiking, bursting, and frequency adaptation—laying a foundation for energy-efficient, event-driven, and highly parallel neuromorphic systems. Compared to conventional artificial neural networks (ANNs) that rely on continuous signal transmission and extensive matrix operations, SNNs utilize discrete spikes for communication, enabling sparse, asynchronous processing that enhances robustness and adaptability in real-world sensory tasks while reducing energy consumption [28,29,30]. Moreover, the introduction of memristive elements into neuromorphic sensory frameworks has driven substantial progress in domains such as sensory processing, adaptive learning, and cognition. For instance, coupling memristors with multimodal sensors enables the emulation of biological sensory modalities such as vision, touch, and olfaction, expanding their role in neuromorphic perceptual systems. Such hybrid architectures can transduce external stimuli into temporal spike streams and execute near-sensor preprocessing, mimicking the behavior of sensory neurons like retinal or somatosensory cells. Memristors’ capacity to perform direct spike encoding from sensory inputs without extensive digital preprocessing renders them ideal for edge AI deployments, such as robotics, wearables, and bio-implantable interfaces.

Despite notable progress in various aspects, memristor-based neuromorphic systems still face several challenges for large-scale deployment, such as significant device variability, limited endurance, and stochastic switching behaviors, all of which may compromise the computational accuracy and scalability. Furthermore, efficient learning algorithms that account for the non-ideal characteristics of memristors are still under active development. Ongoing efforts in approximate computing, CMOS–memristor hybrid design, and the co-optimization of algorithms and hardware aim to overcome these obstacles and accelerate the transition toward practical applications.

In this review, we present a comprehensive assessment of memristors in enabling spiking neuromorphic systems targeting brain-like perception and computation. The discussion begins with the biological basis of neural spiking and common computational models, followed by the underlying switching physics of memristors and their utility in mimicking neuronal dynamics. We then summarize key developments in spiking neuron circuit designs based on memristors, highlight applications in vision, touch, and multimodal bioinspired sensing, and conclude by identifying major challenges and future perspectives.

While several prior reviews have examined memristors for neuromorphic computing, most focus predominantly on synaptic functionalities or general device mechanisms without emphasizing spiking neuron implementations. Furthermore, relatively few works systematically analyze how TSMs uniquely enable diverse spiking neuron behaviors—such as leaky integration, oscillations, stochastic firing, and action potential-like dynamics—at the device and circuit levels. Moreover, the interplay between memristive neuron models and multi-modal sensory systems (e.g., vision, touch, and olfaction) remains underexplored.

This review seeks to bridge these gaps by providing a focused, structured, and critical evaluation of memristor-based spiking neuromorphic systems, encompassing device mechanisms, circuit implementations, and bio-inspired perception applications. In doing so, it offers a unified perspective on how TSM-based neurons can be harnessed for energy-efficient, near-sensor computing, thereby advancing the state-of-the-art in neuromorphic engineering.

## 2. Biological Basis and Computational Models of Spiking Neurons

**Figure 1:**
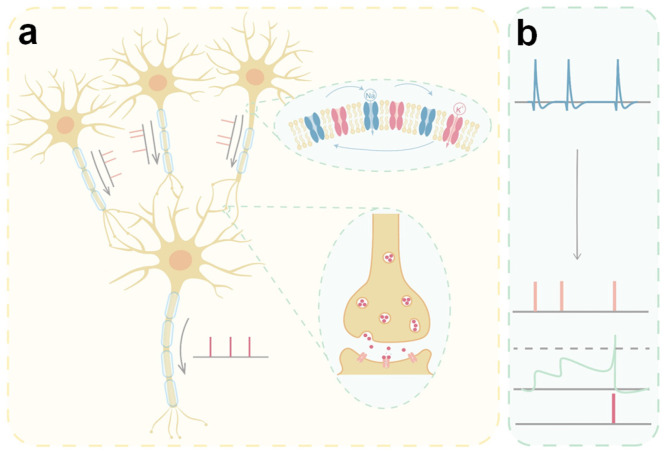
(**a**) Schematic illustration of the connectivity between neurons. (**b**) Schematic of action potential integration within a neuron.

Biological neurons encode and transmit information through discrete electrical spikes, known as action potentials, rather than the clock-synchronized binary levels of digital logic [31]. This spike-based signaling is inherently sparse, asynchronous, and event-driven, underpinning higher-order brain functions such as perception, memory, and motor control. Anatomically, a neuron typically possesses thousands of dendritic spines, a soma with a membrane capacitance of approximately 200 pF, and a myelinated axon capable of propagating action potentials at velocities of 1–120 m/s [32,33]. As illustrated in Figure 1a, dendrites receive inputs from presynaptic neurons and can exhibit nonlinear phenomena—such as NMDA-mediated local spikes and calcium-induced calcium release [34,35]. The soma integrates these inputs and determines whether the membrane potential exceeds the firing threshold (Figure 1b). When this threshold is crossed, an action potential is generated at the axon hillock and propagates along the axon to the synaptic terminals, triggering neurotransmitter release and downstream signaling. Voltage-gated ion channels (Na^+^ and K^+^ types) coordinate depolarization and repolarization, shaping the spike waveform and timing [36,37,38,39].

Biological neurons display diverse spiking patterns to fulfill various functional roles in the brain. This functional diversity underpins the brain’s capacity for temporal encoding, information compression, and stimulus discrimination. Replicating these firing patterns in hardware implementations is essential for achieving biologically faithful neuromorphic architectures. Thus, a thorough understanding of the electrophysiological mechanisms underlying neuronal excitability and information coding is crucial for advancing neuroscience and designing future spike-based hardware systems.

The Hodgkin–Huxley (H-H) model provides a quantitative framework for describing the biophysical mechanisms underlying action potential generation in neurons [40]. It models the neuron as an equivalent electrical circuit composed of capacitive, resistive, and electromotive components, corresponding, respectively, to the lipid bilayer membrane, ion channels, and ionic reversal potentials. The membrane potential dynamics are governed by a set of nonlinear differential equations that capture the voltage- and time-dependent behavior of sodium (Na^+^) and potassium (K^+^) channels, along with a passive leak conductance. The primary equation is given by:

(1) $$C_{m}\frac{dV_{m}}{dt}=-\left[g_{Na}m^{3}h\left(V_{m}-E_{Na}\right)+g_{K}n^{4}\left(V_{m}-E_{K}\right)+g_{L}\left(V_{m}-E_{L}\right)\right]+I_{ext}$$

where *C_m_* is the membrane capacitance, *V_m_* is the membrane potential, and *g_Na_*, *g_K_*, and *g_L_* denote the maximal conductances of Na^+^, K^+^, and leak channels, respectively. The variables *m*, *h*, and *n* represent gating variables that determine the probability of channel opening. This formalism enables the model to reproduce key neuronal phenomena including threshold firing, spike initiation, refractory periods, and subthreshold oscillations.

While the H-H model provides a highly detailed and biologically realistic simulation of neuronal behavior, its computational complexity is significant. Each time-step simulation typically requires around 1200 floating-point operations, making it computationally expensive and limiting its scalability for large networks or hardware implementations. This high computational cost presents challenges for real-time applications, particularly in neuromorphic systems where low power and efficiency are crucial. To reduce the computational cost while retaining biologically relevant timing, simplified neuron models have been proposed. Notably, the LIF model has become the predominant choice in theoretical neuroscience and neuromorphic implementations [41,42,43]. In the LIF model, the membrane potential integrates the input current over time and triggers a spike once a threshold is crossed, followed by a reset mechanism. While not as biologically detailed as the H-H model, the LIF model strikes a pragmatic balance between biological relevance and computational efficiency, making it particularly advantageous for designing large-scale SNNs [44]. Typical LIF implementations run in digital logic at <10 FLOPs/spike or in analog CMOS at <100 pJ/spike [45,46].

Beyond the LIF model, several other neuron models—such as the Izhikevich, FitzHugh–Nagumo (FHN), and Hindmarsh–Rose models—have been introduced to capture more complex neuronal dynamics. The two-variable Izhikevich model matches 20 neuronal firing patterns at 50× less computation than H-H, while FHN captures excitability class-I/II bifurcation with analytical tractability [47]. For example, the Izhikevich model employs a two-dimensional nonlinear differential equation framework, enabling it to reproduce diverse neuronal firing behaviors—tonic firing, phasic bursting, and rebound spiking—with minimal parameter tuning. The FHN and Hindmarsh–Rose models abstract neuronal excitability into low-dimensional dynamical systems, facilitating the exploration of dynamic phenomena such as bifurcation and chaos [47,48,49].

In conclusion, spiking neuron modeling serves as a critical bridge between biological neuroscience and AI system design. Ranging from the intricate ion–channel kinetics of the H-H model to the computationally efficient abstractions of the LIF and Izhikevich models, the field is evolving toward neuron models that balance biological fidelity with hardware implementability [50]. This foundation underpins both the understanding of cerebral function and the ongoing innovation in neuromorphic computing technologies.

Selecting appropriate spiking neuron models hinges on balancing biological realism and computational efficiency. Therefore, in neuromorphic engineering—where energy efficiency and scalability are paramount—identifying suitable hardware-level implementations of neuron models can significantly simplify computation. Memristors, with their inherent hardware compatibility and intrinsic nonlinearities that emulate ion–channel dynamics, emerge as promising candidates for implementing diverse spiking behaviors in low-power, compact hardware systems. When implemented with memristors, these simplified models benefit from the inherent characteristics of memristive devices, such as threshold-switching and nonlinear behavior, to replicate the action potential dynamics seen in biological neurons. The trade-offs between these models in terms of biological fidelity versus hardware implementation are critical for choosing the right model for a given application. For example, while the H-H model offers high fidelity, it is challenging to implement efficiently in low-power neuromorphic systems due to its high computational demand.

## 3. Memristor Fundamentals for Neuromorphic Applications

In 1962, negative differential resistance (NDR) was first observed in metal–oxide sandwich structures based on Al_2_O_3_, ZrO_2_, TaO_X_, and TiO_2_, laying the conceptual foundation for subsequent memristor research [51]. The memristor concept itself was formally introduced by Chua in 1971, who defined it as the fourth fundamental passive circuit element, complementing the resistor, capacitor, and inductor [52]. Owing to their intrinsic nonlinear switching dynamics, memristors have since drawn extensive interest within the neuromorphic engineering community as promising building blocks for bio-inspired neural systems. A typical memristor comprises a metal–insulator–metal (MIM) sandwich structure, in which the dielectric layer’s resistance modulates in response to the applied voltage or current. A variety of materials are used for the dielectric layer, including transition metal oxides (e.g., TiO_2_, HfO_2_, and TaO_X_), chalcogenides, and perovskite compounds. Depending on whether the conductance state is retained, memristors are categorized into nonvolatile and TSMs [53,54,55,56,57,58,59]. Nonvolatile memristors act as programmable synaptic weights, retaining conductance states without power [42,60,61,62,63], while volatile TSMs are essential components in neuromorphic systems, especially for emulating the spiking behavior of biological neurons.

**Figure 2:**
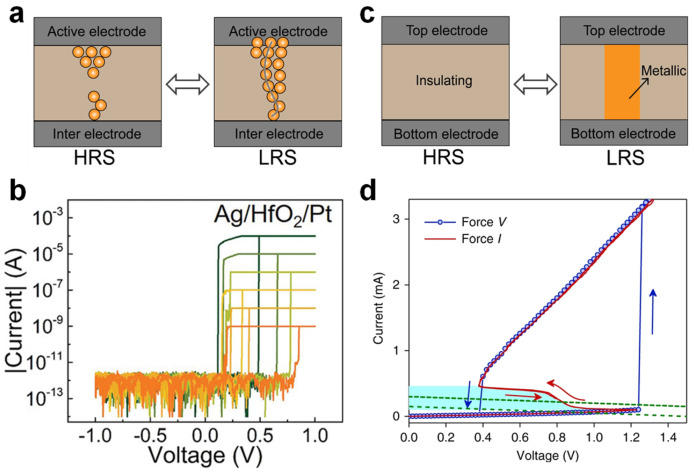
(**a**) Redox-type switching mechanism. (**b**) Typical I–V characteristics of a redox-type volatile threshold-switching memristor (TSM) under different current compliance levels, with each color corresponding to a specific compliance setting. (**c**) Mott-transition mechanism. (**d**) Typical I–V characteristics of a VO_2_ Mott TSM. Image (**c**) is reproduced with permission [65]. Copyright 2020, Wiley-VCH. Image (**d**) is reproduced with permission [63]. Copyright 2018, Springer Nature.

TSMs used in neuron circuit design are mainly classified into two types based on their switching mechanisms: electrochemical redox and Mott memristors [60,61,64]. As shown in Figure 2a, redox memristors typically incorporate active electrodes (e.g., Ag or Cu) and oxide-based dielectrics (e.g., HfO_2_ or TaO_X_) and operate via electrochemical reactions and metal ion migration. When an external electric field is applied, metal atoms at the active electrode are oxidized and migrate across the dielectric, forming a metallic filament that connects both electrodes—a SET operation that switches the device from the high-resistance state (HRS) to the low-resistance state (LRS) [65], as depicted in Figure 2b. Once the bias is removed or sufficiently lowered, the conductive filament ruptures spontaneously to minimize the interfacial energy, thereby restoring the device to HRS and completing a reversible switching cycle. Experimental studies show that these devices typically exhibit switching times in the order of tens of nanoseconds; the HRS current can also drop as low as 1 pA [65,66,67,68]. A compliance current (CC) is typically applied during the testing of redox-type memristors to prevent permanent thermal damage to the conductive filament, which would otherwise hinder spontaneous rupture. These devices benefit from structural simplicity, mature process compatibility, low standby power, and scalability, but their switching behavior can be highly variable due to stochastic filament growth, leading to cycle-to-cycle resistance scatter, which presents challenges for reliable neuromorphic hardware implementations [69,70,71,72,73,74].

Mott memristors, depicted in Figure 2c, based on correlated electron materials like VO_2_ and NbO_X_, exhibit reversible insulator-to-metal transitions (IMT) driven by temperature or electric field changes [65,75]. As shown in Figure 2d, the I–V curve of a Mott-type TSM demonstrates the characteristic transition from insulating (HRS) to metallic (LRS) states when the applied voltage exceeds a threshold [63]. Mott-based TSMs exhibit fast, stable switching with a characteristic switching time of approximately a few nanoseconds. These devices are capable of replicating rapid spiking behaviors observed in biological neurons. However, the high instantaneous current required for switching the mA range contributes to a higher power consumption. This makes Mott-TSMs suitable for high-speed neural circuits, but presents challenges for low-power neuromorphic systems where energy efficiency is critical [76].

Researchers have implemented a variety of TSM-driven neuron circuits to emulate diverse spiking patterns, including oscillatory [72], LIF, stochastic [42,62,66,77,78], and frequency-adaptive firing behaviors [63]. The most common is the LIF type, where a TSM is connected in parallel with a capacitor to accumulate charge and trigger spiking upon reaching a threshold, followed by a rapid reset. For instance, LIF neurons with tunable firing frequencies and refractory periods have been realized using materials such as SiO_2_, TaO_X_, and NbO_X_. Oscillatory neurons utilize the NDR region of TSMs coupled with passive RC networks to generate periodic spiking under constant bias, suitable for simulating rhythmic neuronal activities. Moreover, researchers have exploited intrinsic thermal and conductance fluctuations in devices to build stochastic neurons that emulate probabilistic firing for applications such as Boltzmann machines. The Hodgkin–Huxley (H-H) neuron model, representing detailed ion channel dynamics, can also be implemented using arrays of TSMs to mimic Na^+^/K^+^ transport phenomena. Pickett et al. utilized two NbO_X_ TSMs to replicate the dynamic currents of Na^+^ and K^+^, producing multi-phase spiking and membrane potential oscillations. Subsequent studies introduced programmable memristors like ECRAM combined with TSMs to build adaptive neuron circuits with tunable spiking dynamics, extending the functional emulation of biological neurons. Overall, neuron circuits built upon TSMs enable biologically faithful, energy-efficient, and miniaturized hardware architectures, showing strong potential as core elements in future neuromorphic processors and brain-like perception platforms.

## 4. Memristor-Based Spike Neuron Implementation Scheme

A key goal of neuromorphic engineering is to design compact, energy-efficient electronic elements that accurately emulate the spiking dynamics of biological neurons. Among emerging devices, TSMs provide a distinctive approach to emulate spiking neuron functionalities. Leveraging their intrinsic nonlinear dynamics, spontaneous threshold switching, and time-dependent behavior, researchers have constructed numerous artificial spiking neurons. This section presents a taxonomy of key neuron circuit architectures, implementation strategies, and performance metrics.

### 4.1. Oscillatory Neurons

Oscillatory neurons emulate the rhythmic firing behavior observed in biological neurons by generating periodic spikes when driven by either constant voltage or current inputs. This neuron type is particularly suitable for simulating synchronized and rhythmic neuronal activity, commonly found in neuronal oscillations.

**Figure 3:**
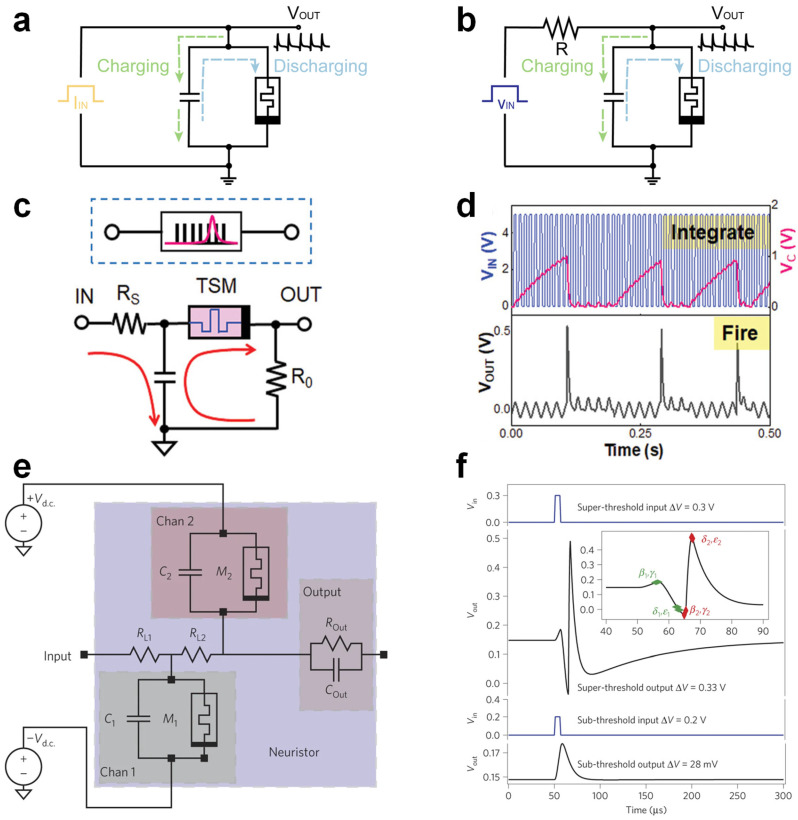
TSM-based neuron circuits. (**a**) Circuit diagrams of oscillatory neurons driven by current and (**b**) voltage, respectively. (**c**) Leaky integrate-and-fire (LIF) neuron circuit employing a TSM device and resistive elements. (**d**) Experimental response of the LIF circuit showing clear integrate-and-fire behavior. (**e**) Equivalent circuit of the Hodgkin–Huxley (H-H) neuron model. (**f**) All-or-none firing behavior of the neuron. Images (**c**,**d**) are reproduced with permission [79]. Copyright 2022, Wiley-VCH. Images (**e**,**f**) are reproduced with permission [64]. Copyright 2013, Springer Nature.

In a current-driven oscillatory neuron circuit, as illustrated in Figure 3a, a TSM is connected in parallel with a capacitor. When the memristor remains in an HRS, the injected current progressively charges the capacitor, gradually elevating the output voltage (V_OUT_). Upon reaching the threshold voltage (V_TH_), the memristor abruptly transitions to an LRS, causing the rapid discharge of the capacitor and a sharp voltage drop. As the voltage drops below the hold voltage (V_HOLD_), the memristor returns to HRS, initiating another charging–discharging cycle and thus producing repetitive spiking. The spike frequency linearly increases with the input current intensity, providing tunable oscillation dynamics analogous to biological neurons. In contrast, the voltage-driven oscillatory neuron circuit (Figure 3b) comprises a memristor, resistor, and capacitor. With a constant voltage applied, the capacitor gradually accumulates charge through the resistor. Once the voltage across the memristor surpasses its threshold, the memristor transitions from HRS to LRS, rapidly discharging the capacitor and causing a sharp voltage drop. After discharging, the voltage falls below V_HOLD_, resetting the memristor to HRS, and the charging cycle repeats. This mechanism also generates rhythmic spiking, with a frequency proportional to the applied voltage, enabling precise control of the spiking patterns.

These memristor-based oscillatory neurons have been extensively applied in neuromorphic computing tasks. Gao et al. demonstrated a self-oscillating neuron based on Pt/NbO_X_/Pt devices with a tunable frequency [80], while Shi et al. further simplified the circuit by utilizing intrinsic parasitic capacitances, significantly reducing the complexity and footprint of oscillatory neuron circuits [67]. Oscillatory neuron models based on TSMs, offer intrinsic spiking behavior driven by volatile resistance changes, eliminating the need for external timing circuits. This makes them highly attractive for compact, energy-efficient neuromorphic systems. However, several limitations remain. First, the high-temperature operation required for Mott-transition devices like VO_2_ raises concerns for system-level thermal management and CMOS compatibility. Second, oscillatory neurons often exhibit poor controllability in the spiking phase, making them less suitable for tasks requiring precise spike timing, such as temporal coding or coincidence detection. Additionally, frequency encoding is inherently analog and can suffer from noise sensitivity, limiting robustness under fluctuating inputs. Some designs also rely on external passive components (e.g., capacitors or resistors) to tune the oscillation behavior, which increases the area and integration complexity.

### 4.2. Leaky Integrate-and-Fire (LIF) Neurons

Beyond periodic spiking, the neural firing mechanism follows a principle of temporal integration: if multiple synaptic inputs collectively exceed the threshold within a certain time window, a spike is fired; otherwise, the integrated input gradually attenuates. This behavior is typically modeled by the LIF neuron, which captures both integration and leakage processes. The “leaky” membrane potential of neurons is analogous to the conductivity decay in volatile memristors, which reflects a key dynamic aspect of memory decay. The volatility of memristors allows artificial neurons to autonomously return to the resting potential after firing, or revert to a quiescent state if the input is insufficient to trigger firing. As shown in Figure 3c, the TSM-based LIF neuron circuit consists of a TSM in series with a resistor R_0_ and in parallel with a capacitor C. Input pulses are applied through resistor R_S_, and the voltage across R_0_ is monitored as the spike output. When a series of voltage pulses is applied, the capacitor charges, causing a gradual increase in its voltage—this process is termed “integration”. When the TSM voltage exceeds V_TH_, it transitions from a high-resistance to a low-resistance state. Since the off-state resistance of the TSM is much larger than R_0_, the voltage drop across R_0_ remains near zero during charging. Upon threshold activation, the TSM rapidly drops to low resistance, causing a voltage spike across R_0_ until the device reverts to the off-state as the voltage falls. Since the discharge loop has a lower resistance, a sharp voltage spike is observed across R_0_, denoting the “firing” event (Figure 3d). The spiking rate grows with stronger inputs and diminishes with increasing RC values in the LIF circuit.

Zhang et al. developed a LIF neuron based on Ag/SiO_2_/Au TSMs, demonstrating adjustable firing rates and refractory periods [66]. Building upon these foundational neuron circuits, subsequent research expanded their application into more comprehensive neuromorphic architectures. For instance, Wang et al. further proposed a fully memristive artificial neural network integrating threshold-switching neurons with non-volatile memristor synapses, successfully demonstrating unsupervised learning and pattern recognition capabilities [42]. Such neuron–synapse integrated architectures underscore the feasibility of memristor-based neurons in practical neuromorphic computing tasks, highlighting their potential for scalable brain-inspired computational systems. Yuan et al. employed VO_2_-based memristors to construct asynchronous spike encoders that transform multichannel biosignals, such as EEG and ECG, into sparse spiking sequences, thereby reducing the data size and power demand [81]. Furthermore, memristor-based LIF and ALIF neurons implemented using VO_2_ devices were incorporated into a long short-term memory spiking neural network (LSNN) to enable efficient spike-coded signal processing. The system achieved remarkable classification accuracy—95.83% for arrhythmia and 99.79% for epilepsy detection—validating the capability of VO_2_ memristors in enabling scalable and efficient neuromorphic platforms for biomedical signal analysis. Compared to CMOS LIF neurons, TSM-based implementations offer significantly reduced energy per spike, as well as area advantages due to simpler circuitry. However, current designs often suffer from limited linearity and the poor control of leakage currents, which can reduce the spike-timing precision. Further studies should focus on stabilizing integration behavior through selector-assisted topologies or hybrid analog-digital feedback loops.

### 4.3. Hodgkin–Huxley (H-H) Neurons

H-H neurons represent one of the most biologically realistic models in computational neuroscience, capable of reproducing a wide range of electrophysiological behaviors such as action potentials, thresholding, and refractory dynamics. Implementing H-H-type neurons with memristive elements requires fine-grained control over ionic conductance, which can be achieved through TSMs that emulate the gating dynamics of voltage-dependent ion channels. A pioneering example was demonstrated by Pickett et al., who utilized Mott-type memristors to mimic the dynamic behaviors of Na^+^ and K^+^ channels [64]. Their circuit incorporated two TSMs with opposing polarities, regulated by separate DC voltage sources, to replicate the bidirectional flow of ionic currents—analogous to the depolarization and repolarization processes in biological membranes (Figure 3e). The resulting waveform reproduced key features of biological action potentials, including depolarization, hyperpolarization, and refractory periods (Figure 3f). This work marked a significant step toward the physically accurate emulation of spiking dynamics in hardware.

However, while the implementation achieves high fidelity in replicating the H-H model, it also exhibits substantial complexity and overhead. The requirement for multiple discrete components—including separate voltage sources, resistors, and capacitors—limits its scalability and integrability in dense neuromorphic circuits. Additionally, the relatively high power consumption and limited dynamic range of Mott memristors present challenges for practical edge-AI applications that demand energy efficiency and robustness under variability. From a computational perspective, the detailed biophysics modeled by H-H neurons are often overkill for many real-world tasks, where simpler models such as LIF or adaptive spiking suffice with significantly less hardware complexity. Nevertheless, H-H-type implementations are valuable for benchmarks, biological validation, and exploratory modeling, especially when studying complex ion–channel interactions, pharmacological responses, or neuron-level pathologies.

Looking forward, future H-H-inspired implementations may benefit from compact, hybrid architectures that integrate memristive dynamics with programmable analog/digital modules. This approach could preserve key nonlinear behaviors while reducing the circuit size and improving the power efficiency. Additionally, material engineering in Mott systems—for example, by controlling phase transition thresholds or improving the thermal stability—could further enhance the reproducibility and endurance of these neurons in large-scale neuromorphic platforms.

### 4.4. Stochastic Neurons and Origins of Randomness in Memristors

The stochastic behavior of memristors is one of the intriguing characteristics that can play a significant role in neuromorphic computing systems, particularly in randomness-based applications such as true random number generation (TRNG). Stochastic switching in memristors primarily originates from atomic migration, thermal noise, and electron correlation effects, varying by the material type. In redox-based memristors, the movement of metal ions under an applied electric field is a key factor in generating stochasticity. This migration leads to the formation of conductive filaments in the dielectric material, and the random nature of this filament growth contributes to variability in the device’s resistance. As the SET and RESET operations occur, the resistance states can fluctuate due to the inherent randomness in atomic migration. In Mott memristors, stochastic switching originates from critical electron–electron interactions and phase transition dynamics, which are highly sensitive to local temperature and electric field gradients. These phenomena result in observable cycle-to-cycle and device-to-device variations in the switching delay, threshold voltage, and retention characteristics.

The inherent stochasticity in memristive devices, particularly in TSMs, offers a valuable mechanism to emulate the probabilistic behavior of biological neurons. Unlike deterministic neurons, which fire in response to precise input conditions, biological neurons often exhibit variable firing thresholds and spike timing due to thermal fluctuations and ion channel noise. Emulating such stochasticity is critical for realizing probabilistic computation, Bayesian inference, and uncertainty quantification in neuromorphic systems.

This stochasticity, while traditionally viewed as a source of unreliability, can be purposefully exploited. For instance, Miao et al. developed an electronic stochastic neuron using a CuS/GeSe-based TSM, capable of mimicking random firing behavior observed in biological neurons and enabling Bayesian inference within a spiking neural network (SNN) [82]. The stochastic neurons markedly decreased the fatal misjudgment rate in tumor classification scenarios, a typical shortcoming in traditional ANN models. Compared with SNNs built from deterministic neurons, this stochastic neuron approach improved the uncertainty estimation accuracy, boosting the confidence evaluation by 81.2%. This study highlights the potential of integrating stochastic memristors into neuromorphic systems, paving the way for efficient hardware-based probabilistic computing. Mao et al. reported a stacked IGZO-based TSM neuron with the sigmoid firing probability, mimicking the probabilistic response characteristics of natural neurons [83]. This stacked TSM architecture exhibited reduced relative switching variability (6.8%) compared to single-device implementations (59%), critical for deep Boltzmann machines where the weight noise severely degrades the log-likelihood. Moreover, the IGZO stochastic neuron was applied in probabilistic unsupervised learning for handwritten digit reconstruction using a restricted Boltzmann machine, achieving a 91.2% recognition accuracy. This IGZO-based stochastic neuron, with its repeatable probabilistic firing, emulates brain-like probabilistic computation, offering significant implications for hardware SNNs in sensory processing, motor control, and reasoning.

Beyond probabilistic neural modeling, the stochastic behavior of TSMs is also suitable for hardware-based TRNGs. Phase-change neurons, as demonstrated by Tuma et al., leverage intrinsic thermal noise and threshold dispersion for robust random spike generation, offering potential for secure neuromorphic encryption and stochastic optimization [78]. These developments highlight the duality of memristor stochasticity—as both a physical constraint and a computational asset.

These studies demonstrate the promise of stochastic memristive neurons in probabilistic inference and unsupervised learning tasks. Nonetheless, these models face notable limitations. The reliance on physical noise sources introduces significant variability not only across devices, but also across time in the same device, making consistent spike-rate modulation and task-specific calibration challenging. Moreover, stochasticity in spike generation, while useful for probabilistic inference, can undermine the performance in applications requiring deterministic or time-locked responses, such as sequence learning or temporal pattern recognition. Additionally, the mapping from input stimuli to desired firing probabilities often lacks linearity or interpretability, complicating training and co-optimization with learning algorithms.

Compared to LIF and oscillatory neurons, stochastic memristive neurons provide a valuable mechanism for modeling neural noise and probabilistic computation, but their application scope is more suited to ensemble or redundancy-based networks rather than precision-centric tasks. Future work may benefit from integrating tunable stochasticity (e.g., a bias-controlled noise injection) or leveraging noise-aware learning frameworks such as contrastive divergence or spike-based variational inference to enhance the performance and system stability.

Looking forward, engineering stochasticity at the device level through the material design, geometry optimization, and temperature control could enable tunable randomness, paving the way for neuromorphic hardware platforms with adaptive uncertainty handling. Moreover, hybrid architectures combining deterministic and stochastic neurons may allow for biologically inspired trade-offs between precision and flexibility in spiking neural networks. In summary, the stochastic properties of memristors—once considered detrimental—are increasingly being harnessed to emulate the noisy dynamics of biological neurons. This opens new avenues for energy-efficient probabilistic computing and robust neuromorphic perception systems, especially in real-world environments where uncertainty and variability are inherent.

### 4.5. Complex Spiking and Reconfigurable Neurons

Beyond the fundamental neuron types, complex firing patterns including burst firing, frequency adaptation, and neuronal inhibition are also critical for enabling energy-efficient neural computation. Yi et al. demonstrated over twenty spiking behaviors using VO_2_ memristors, such as bursting, frequency adaptation, and subthreshold oscillations, as well as characterized capacitor-dependent mechanisms and stochastic phase-locked firing, highlighting the dynamic richness and computational complexity of biological neurons [63]. Kim et al. proposed an artificial neuron architecture based on NbO_X_ memristors, utilizing metal–insulator phase transitions and thermodynamic effects in flexible organic substrates to replicate 18 biological firing patterns, including frequency adaptation and subthreshold oscillations [84]. By leveraging thermal coupling, the system achieves neuron-to-neuron communication through the heat flow alone, bypassing traditional interconnections and greatly reducing the energy consumption. The experimental results showed that this architecture achieved over 10^6^-fold energy savings compared to conventional digital processors in graph optimization tasks, demonstrating the great potential of thermodynamics in neuromorphic computing. Yu et al. proposed a memristor-based reconfigurable spiking neuron architecture capable of emulating multiple firing patterns at the hardware level, including fast spiking, adaptive spiking, phase spiking, and bursting. [85] The neuron design integrates NbO_X_-based spike units with electrochemical memristors (ECRAMs), enabling the programmable switching of firing behaviors by adjusting the ECRAM resistance, thus achieving function-level reconfigurability without altering the circuit structure. Such a resistance-coded polymorphism paves the way for compiler-directed neuron “microcodes”, enabling run-time reconfiguration analogous to micro-op fusion in CPUs.

While a range of memristor-based neuron implementations—from redox and Mott devices to TaO_X_, NbO_X_, and VO_2_ technologies—have been demonstrated, their power consumption, spiking frequency, endurance, and switching-energy characteristics vary widely. Table 1 summarizes these key experimental metrics—the power consumption, switching energy, operating frequency range, spiking rate, and endurance—which are critical for assessing the feasibility of large-scale, energy-efficient neuromorphic systems.

In summary, TSMs provide a solid hardware foundation for building compact, low-power, and biologically plausible spiking neurons due to their pronounced nonlinear switching properties. By integrating TSMs with passive elements such as capacitors and resistors, various spiking behaviors—including oscillatory, LIF, stochastic, and H-H types—have been successfully replicated, with support for reconfigurable neuron implementations. These efforts not only greatly reduce the circuit complexity, but also demonstrate excellent energy efficiency and integration potential, advancing neuromorphic computing from basic units to multifunctional and scalable systems. Memristors offer unparalleled advantages in hardware-level spike encoding, probabilistic reasoning, and multimodal neural dynamics, laying the groundwork for next-generation intelligent and adaptive brain-like systems.

## 5. Memristor-Based Spiking Neuromorphic Perception

Humans rely on photoreceptors, thermoreceptors, mechanoreceptors, and olfactory receptors to receive physical signals from the external environment, which are encoded into spikes and transmitted to neural networks for perception and learning [88,89]. Such systems exhibit exceptional capabilities in extracting and encoding environmental cues, allowing energy-efficient, adaptive, and resilient cognitive functions [90,91]. Inspired by this, memristor-based neuromorphic perception systems integrate spiking neurons with sensory components to achieve direct hardware-level encoding and preprocessing, building compact, low-power platforms with real-time and edge computing capabilities [92,93]. In this section, we focus on recent developments and bio-inspired applications of spiking perception systems based on memristive neurons.

### 5.1. Spiking Visual Perception Systems

With the rapid development of intelligent robotics, autonomous driving, and wearable electronics, the demand for artificial vision systems with high efficiency, low-power consumption, and real-time responsiveness is growing rapidly [94,95,96,97]. Biological visual systems exhibit exceptional efficiency and adaptability, largely attributable to their massively parallel architectures, event-driven signaling mechanisms, and sparse neural encoding strategies. In particular, retinal networks perform robust front-end preprocessing by filtering out irrelevant visual noise and extracting salient features in real time. This capability substantially reduces the computational burden on downstream neural circuits, enhancing the overall energy efficiency and response speed, thus providing a compelling model for bio-inspired visual perception systems [86,98,99,100]. Consequently, the development of biologically analogous artificial vision systems, especially those that enable direct optical-to-spike conversion and local signal preprocessing in hardware, is of great significance for building cognitive brain-inspired intelligent systems and constitutes a critical direction for achieving next-generation high-efficiency intelligent perception.

**Figure 4:**
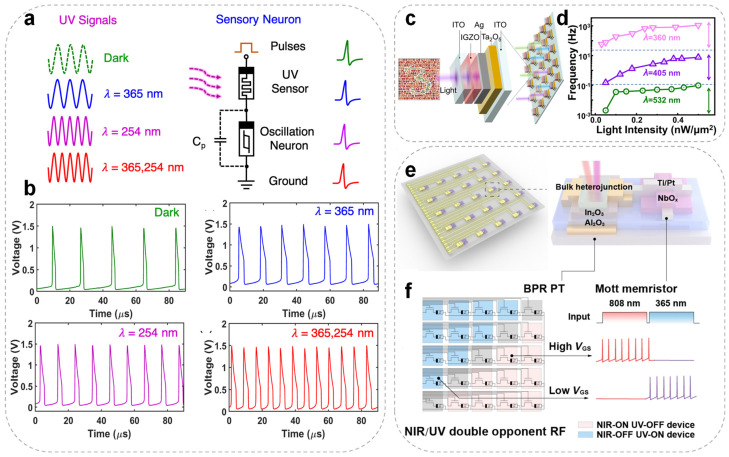
(**a**) Schematic illustration of various ultraviolet (UV) signals (left) and the artificial sensory neuron. (**b**) Experimentally observed neuronal spiking at four distinct frequencies under different UV stimuli in the oscillatory neuron. (**c**) Structural diagram of the artificial spiking cone photoreceptor (VISCP) array based on an ITO/TaO_X_/Ag/IGZO/ITO stack. (**d**) Spike frequency response curves of the VISCP array showing wavelength-dependent selectivity. Reproduced with permission. (**e**) Schematic of an artificial visual spiking neuron composed of a synaptic phototransistor (BP RPT) and an NbO_X_ memristor. (**f**) V_GS_ tuned spiking neuron array configured as a directional NIR–UV DORF module for parallel spike encoding and signal preprocessing. Images (**a**,**b**) are reproduced with permission [86]. Copyright 2020, American Chemical Society. Images (**c**,**d**) are reproduced with permission [73]. Copyright 2023, Springer Nature. Images (**e**,**f**) are reproduced with permission [101]. Copyright 2025, Springer Nature.

In the biological visual system, retinal photoreceptors detect and integrate optical signals and transmit them to the brain’s visual cortex. Figure 4a illustrates an artificial visual sensory neuron developed by Wu et al., consisting of an InGaZnO_4_ (IGZO_4_) ultraviolet (UV) photoreceptor connected in series with a NbO_X_-based TSM, designed to emulate functionalities of the human visual system [86]. When a beam of UV light is vertically incident on the photoreceptor, the UV stimuli of different wavelengths effectively modulate the resistance state of the IGZO_4_ photodetector. Consequently, the voltage across the series-connected NbO_X_ spiking neuron also varies, leading to the generation of spike signals at different frequencies, as shown in Figure 4b. This artificial visual neuron thus exhibits light-modulated neuronal behavior, with its spiking rate dependent on the wavelength of the incident UV light. The contraction and relaxation of ciliary muscles enable the eye to focus on objects at varying distances. A TaO_X_ memristor-based photoelectric spiking neuron system has recently been developed to replicate the depth perception function of biological vision [79]. By integrating a photoresistor with a spiking neuron circuit, the system encodes optical stimuli from different distances into distinct spike frequency sequences, thereby realizing depth sensing and encoding. A binocular vision system was further constructed using this neuron, where spike frequency differences from left and right inputs were processed by an artificial neural network to achieve accurate spatial localization with a recognition accuracy of 90%. This study demonstrates the hardware-level emulation of biological depth perception and provides a practical approach to implementing stereo vision in neuromorphic systems. Wang et al. vertically integrated TaO_X_ memristors with IGZO photodetector layers to develop a spiking cone photoreceptor array (VISCP) (Figure 4c) featuring ultra-low power consumption (≤400 pW), a compact structure, and color-selective artificial visual sensing [73]. VISCP utilizes wavelength-dependent conductance modulation to encode various colors within the visible spectrum into spike frequencies, yielding more than 1.5 orders of magnitude in the frequency distinction (Figure 4d). When integrated into a convolutional spiking neural network (SNN), the system demonstrated a color classification accuracy of 83.2% under variable lighting conditions. These results highlight the VISCP’s potential for both biomimetic vision and intelligent sensing tasks.

Furthermore, Li et al. integrated synaptic phototransistors (BPR PT) with NbO_X_ memristors to construct a spiking neuron array (Figure 4e) with dual-opponent receptive fields and directional selectivity, enabling the first device-level encoding of combined spatial-color information [101]. Figure 4f illustrates how shifting the load line by tuning VGS relative to the two switching thresholds of the TSM enables excitatory or inhibitory spike outputs under NIR or UV illumination. Emulating V1 cortical functionality, the system performs spike responses to color edges using color opponency and directional filters, enhancing the recognition of directional and chromatic variations, and improving SNN reliability in complex scenes under low illumination. Compared to conventional color-sensing approaches, this system significantly boosts front-end feature extraction and information compression, highlighting the promise of neuromorphic vision in edge-aware perception and scene understanding. The research marks a step forward for neuromorphic vision, transitioning from basic color recognition to a deeper comprehension of image structures.

### 5.2. Spiking Tactile Perception Systems

**Figure 5:**
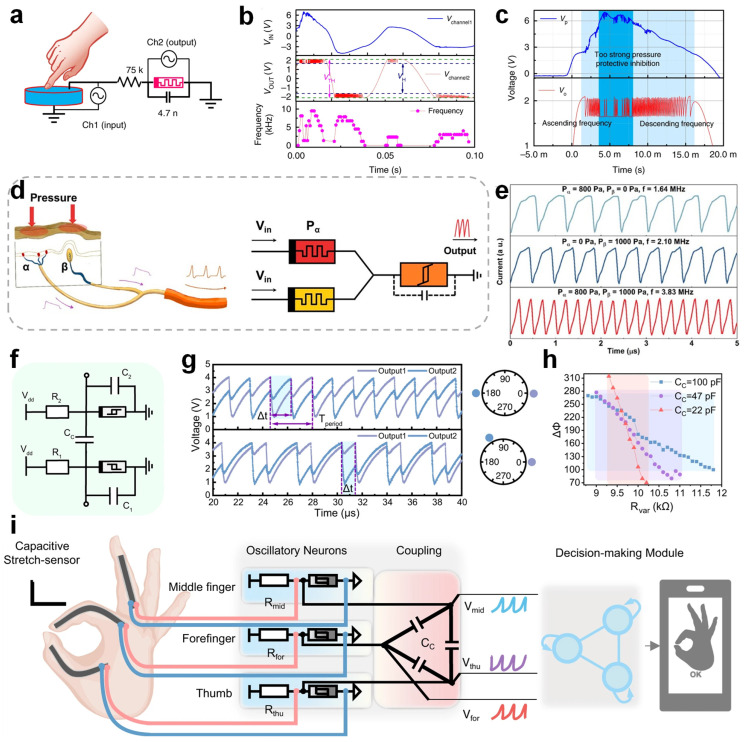
(**a**) Schematic of the circuit architecture for an artificial spiking mechanoreceptor system based on NbOx memristors. (**b**) Output spike signals from the artificial sensing system under varying pressure intensities and (**c**) their corresponding magnified view. (**d**) Schematic illustration of multi-source tactile signal integration, showing how biological tactile inputs from different stimuli are encoded into a single high-frequency spike train, and how this process is emulated by a memristor-based artificial mechanoreceptive system. (**e**) The spiking frequency increases when two pressure stimuli are applied simultaneously. (**f**) Schematic diagram of two capacitively coupled oscillatory neurons. (**g**) Spiking output results showing synchronization patterns and phase differences. (**h**) Curve of phase difference versus resistance under various coupling capacitances. (**i**) Illustration of a gesture recognition system implemented using the oscillatory coupling network. Images (**a**–**c**) are reproduced with permission [104]. Copyright 2020, Springer Nature. Images (**d**,**e**) are reproduced with permission [105]. Copyright 2021, American Chemical Society. Images (**f**–**i**) are reproduced with permission [106]. Copyright 2024, Springer Nature.

In biological systems, tactile neurons like cutaneous mechanoreceptors transduce external pressure into spike signals, which are relayed to the somatosensory cortex for further processing to discern object properties and avoid potentially harmful stimuli [102,103]. Zhang et al. developed an artificial spiking afferent nerve (ASAN) architecture using NbO_X_-based Mott memristors to mimic the sensory function of transforming analog stimuli into spike trains [104]. The system employs the negative differential resistance characteristic of NbO_X_ memristors to construct a compact oscillator circuit capable of generating spike signals with stimulus-dependent frequencies. Under low-to-moderate stimulation, the spike frequency exhibits a near-linear dependence on the stimulus intensity; however, at high intensities, the system displays protective suppression with reduced spiking, mimicking neuronal self-protection. Additionally, the ASAN system was integrated with passive piezoelectric sensors to form a self-powered spiking mechanoreceptor system (Figure 5a) capable of converting mechanical pressure into spikes (Figure 5b,c), highlighting its promise for neurorobotics and brain-inspired sensing. Li et al. constructed an artificial mechanoreceptor system with skin-like characteristics, enabling sustained pressure sensing akin to slow-adapting biological receptors and the enhanced fusion of dual tactile inputs through memristive neuron integration [105]. Figure 5d illustrates the biological mechanism of tactile integration, alongside its artificial counterpart implemented through memristor-based mechanoreceptors. As shown in Figure 5e, when two pressure stimuli are applied simultaneously, the spike frequency increases significantly. The spike frequency after the spatial integration of the parallel sensors is higher than that induced by unilateral stimulation, indicating a notably shortened latency and faster tactile perception. By applying pulse-coupled neural networks (PCNN), this architecture effectively encodes spike frequencies, improves the recognition accuracy, and shows potential in the spatial integration of tactile inputs for intelligent neural interfaces using memristors.

Yang et al. introduced a neuromorphic tactile system based on coupled VO_2_-based memristive oscillators [106]. The schematic illustration of two capacitively coupled oscillatory neurons is shown in Figure 5f. The spiking outputs exhibit clear synchronization patterns, as illustrated in Figure 5g, highlighting their ability to mimic complex tactile processing. The relationship between the phase difference and resistance under various coupling capacitances, as demonstrated in Figure 5h, provides insights into achieving the precise control of neuron synchronization states. Furthermore, this neuromorphic tactile system was successfully implemented in gesture recognition applications, as shown in Figure 5i, achieving high accuracy and low power consumption, which exemplifies its practical relevance for advanced sensory-processing applications in robotics and wearable systems. In addition, a continuous-time dynamical system was implemented for sensory preprocessing, revealing superior metrics in the energy efficiency, area, and latency, which pushes the boundaries of compact nonlinear neuromorphic circuits.

### 5.3. Spiking Thermosensory and Olfactory Perception Systems

In the somatosensory system, thermoreceptors play a critical role in regulating metabolic processes and preventing damage from harmful external stimuli [107,108]. Thermoreceptors encode thermal stimuli into spike trains that are subsequently interpreted by the nervous system as heat sensation. The realization of artificial spiking thermoreceptors (ASTs) may enable new directions in low-power biomimetic thermal sensing. Shi et al. developed an AST based on an Ag/TaO_X_/AlO_X_/ITO memristor, leveraging the temperature-dependent diffusion kinetics of silver ions in the oxide to emulate biological thermal sensing and encoding [67]. Without requiring extra ADC hardware, the AST converts thermal inputs into spike trains with specific frequencies, consuming less than 240 nW. An artificial thermal perception system was constructed using the AST and a pulse-coupled neural network (PCNN), demonstrating effective thermal image edge detection. An ultra-stable NDR memristor using AlAs/In_0.8_Ga_0.2_As/AlAs quantum wells was introduced by Pei et al., offering improved reliability and compatibility for neuromorphic integration [109]. It maintains stable operation at elevated temperatures (up to 400 °C), supporting long-term reliability in extreme environments. Using this device, the researchers implemented an ultra-compact FitzHugh-Nagumo (FN) neuron circuit without external capacitors, successfully replicating nine typical firing behaviors including phase spiking, spike-frequency adaptation, and subthreshold oscillations, significantly simplifying the circuit design and reducing hardware costs. Additionally, the memristor-based SNN showed a 91.74% classification accuracy in the multimodal voltage–temperature perception, validating its potential for robust computation in harsh environments. This work establishes a reliable device platform and feasible approach toward highly energy-efficient and scalable neuromorphic chips. Wang and co-workers developed a neuromorphic platform that mimics real-time biological olfaction by integrating gas sensing, memory, and processing functions [110]. A volatile Pt/Ag/TaO_X_/Pt memristor serves as a LIF neuron that transforms gas-sensor inputs into spiking signals. The output spikes are further transmitted through non-volatile Pt/Ta/TaO_X_/Pt-based memristive synapses to downstream relay neurons for signal processing and pattern recognition. By leveraging frequency-sensitive synaptic plasticity, the system enables accurate gas recognition and categorization.

### 5.4. Spiking Multimodal Perception Systems

**Figure 6:**
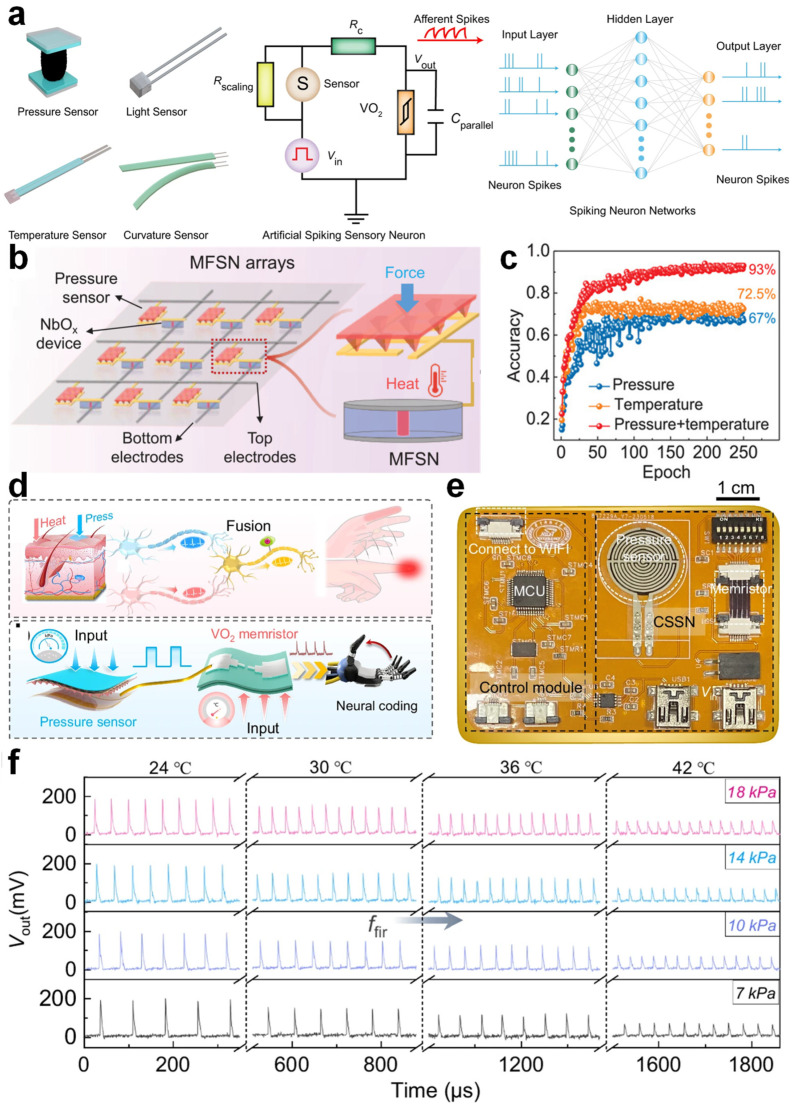
(**a**) Schematic of a neuromorphic multi-sensory system composed of sensors (pressure, light, temperature, and curvature) and artificial neurons, along with an illustration of a spiking neural network (SNN). (**b**) Architecture of the MFSN array based on pressure sensors and NbOx memristors, and schematic of the corresponding multi-sensory neuromorphic computing system. (**c**) Training accuracy versus training epochs under three different modes: pressure, temperature, and multimodal. (**d**) Diagram of a bio-inspired and artificial CSSN-based multi-sensory feedback system. (**e**) Photograph of a flexible integrated sensory feedback system. (**f**) Multi-sensory spiking responses of the CSSN to pressure and temperature stimuli. (**a**) Reproduced with permission [114]. Copyright 2022, Springer Nature. Images (**b**,**c**) are reproduced with permission [115]. Copyright 2022, Wiley-VCH. Images (**d**–**f**) are reproduced with permission [87]. Copyright 2024, Springer Nature.

Building upon the previously discussed unimodal perception systems—covering vision, touch, thermal, and olfactory domains—recent research has emphasized the integration of multiple sensory modalities. In biological somatosensory systems, multimodal integration allows the comprehensive perception of object attributes, enabling precise decision making [88,111,112,113]. Inspired by this, Yuan et al. developed a highly efficient spiking neuromorphic hardware system capable of sensing and encoding various physical stimuli [114]. By integrating VO_2_ TSMs with multiple sensor modalities, the system effectively converts physical signals—such as illumination, temperature, pressure, and curvature—into spike trains. When these spiking outputs were fed into a three-layer spiking neural network, an accuracy of 90.33% was achieved in MNIST-based pressure image classification tasks. Additionally, the neuromorphic sensing modules demonstrated a capability in monitoring the finger curvature for gesture classification, underscoring their significant potential in advanced multimodal neuro-robotic applications. Zhu et al. proposed a heterogeneously integrated multimodal fusion spiking neuron (MFSN) array (Figure 6b), aimed at human-like multisensory perception and object classification [115]. The system converts various sensory inputs like pressure and temperature into spike frequency encoding and performs fusion via memristor-based neuron arrays, effectively mimicking multisensory integration in biological systems. The simulation results indicated that integrating multiple sensory modalities led to a higher recognition rate (93%) in distinguishing cup features compared to using individual modes, with the pressure-only mode achieving 67% and temperature-only mode achieving 72.5%, as shown in Figure 6c. These results demonstrate the feasibility of the MFSN system for advanced robotic intelligence, offering both a high recognition accuracy and energy efficiency.

Li et al. introduced a flexible biomimetic cross-modal spiking neuron (CSSN) based on VO_2_ memristors, capable of the real-time sensing, encoding, and processing of multimodal signals like the pressure and temperature at the hardware level (Figure 6d) [87]. As shown in Figure 6e, the CSSN system is fabricated on a flexible substrate with integrated components including VO2-based memristors, a control module, and a Wi-Fi unit, enabling autonomous sensing and wireless feedback. The VO_2_ memristors employed exhibit an outstanding performance, including >10^12^ endurance cycles, 0.72% cycle-to-cycle variation, 3.73% device uniformity, an <30 ns response time, and bendability to a 1 mm radius. Figure 6f illustrates the multi-sensory response behavior of the CSSN. When subjected to combined pressure (7–18 kPa) and temperature stimuli (24–42 °C), the neuron circuit generates spike trains with distinct firing frequencies, demonstrating effective multimodal sensory encoding. A CSSN-based flexible processing system enables dynamic object recognition with 98.1% accuracy and real-time feedback, highlighting its great potential in wearable human–machine interfaces.

For motion sensing and control, Yang et al. proposed a spike-coding neural circuit driven by emission characteristics, integrating a LiDAR sensor with three H-H neurons based on NbO_X_ memristors for real-time robotic obstacle avoidance [116]. The architecture mimics biologically inspired frequency-modulated distance coding, translating proximity cues into frequency-dependent spike sequences for feedforward decision making without central supervision. Architecturally, the use of memristors enabled compact neuron construction, and the frequency modulation strategy enhanced both reactivity and resilience to environmental changes. By coupling spike frequency patterns with environmental signal representation, this study pioneers a unified neuromorphic strategy for integrated perception and decision making in mobile robots, addressing challenges in energy-efficient autonomous navigation.

These systems embody the paradigm of “perception-as-computation”: encoding and processing occur directly at the signal source, reducing redundancy and latency, suitable for resource-constrained edge AI applications. Nevertheless, challenges remain in device uniformity, heterogeneous integration, and stability; future progress will rely on incorporating plasticity (e.g., STDP), vertical stacking, and 3D crossbar architectures for scalable integration and autonomous perception.

## 6. Conclusions and Outlook

This review has provided a comprehensive analysis of memristor-based spiking neuromorphic systems, highlighting their unique advantages in enabling brain-inspired perception and computation. By accurately replicating essential neuronal behaviors—such as temporal integration, threshold-triggered firing, spike-frequency adaptation, and stochasticity—memristors offer a compact, scalable, and energy-efficient alternative to conventional CMOS-based approaches. Their intrinsic nonlinear switching dynamics, analog conductance modulation, and compatibility with crossbar arrays further allow memristors to serve as both neurons and synapses within integrated SNN platforms.

We systematically summarized the underlying physical mechanisms of TSMs, critically examined representative spiking neuron implementations—including LIF, oscillatory, stochastic, and H-H models—and reviewed their applications across multimodal sensory systems such as vision, tactile sensing, thermosensation, and olfaction. These studies underscore the growing potential of memristor-based hardware in enabling near-sensor, bio-inspired computing at the edge.

Despite these advances, significant challenges persist, notably addressing variability across devices, enhancing the switching endurance, and mitigating timing dispersion in large-scale arrays. These non-idealities manifest as inconsistent thresholds, retention issues, and timing errors, which can compromise temporal precision in spiking networks. In crossbar architectures, sneak-path currents and read/write disturbances further impair long-term stability and energy efficiency. Addressing these limitations demands a multi-level co-optimization strategy—including material innovation (e.g., doped oxides and 2D heterostructures), selector integration, circuit-level compensation, and training algorithms resilient to hardware imperfections. Standardized benchmarking protocols and the statistical modeling of switching kinetics will also be critical for robust large-scale deployment. To advance toward practical implementation, future work should explore novel material stacks and advanced fabrication methods to mitigate device variability and enhance the cycle stability for large-scale integration. Co-design approaches that align device physics, circuit design, and network algorithms will be essential for achieving a real-time, adaptive performance in neuromorphic platforms.

As neuromorphic computing intersects with emerging domains such as intelligent robotics, brain–machine interfaces, and personalized healthcare, demands on low latency, environmental robustness, and on-device learning capabilities will intensify. Memristor-based neuromorphic systems, with their compact form factor and biologically plausible dynamics, could serve as foundational hardware for next-generation cognitive electronics. While broad deployment remains an ongoing challenge, the progress summarized in this review provides both a roadmap and a technical foundation for future innovations in brain-inspired, energy-efficient computation.

## Figures and Tables

**Table 1:** Comparison of TSMs for spiking neurons.

Device	Endurance (Cycles)	Spiking Rate (Hz)	Energy per Spike (J)	Ref.
HfAlO_X_ memristor	>200	1–10	1.6 × 10^−14^	[68]
TaO_X_ memristor	>500	0.1–1200	~50 × 10^−12^	[73]
VO_2_ memristor	>10^6^	~10^5^	~6.9 × 10^−9^	[81]
NbO_X_ memristor	>700	~10^6^	~10^−3^	[86]
Flexible VO_2_ memristor	>10^12^	~10^4^	~4 × 10^−9^	[87]
